# Multiple Source Localization in a Shallow Water Waveguide Exploiting Subarray Beamforming and Deep Neural Networks

**DOI:** 10.3390/s19214768

**Published:** 2019-11-02

**Authors:** Zhaoqiong Huang, Ji Xu, Zaixiao Gong, Haibin Wang, Yonghong Yan

**Affiliations:** 1 Key Laboratory of Speech Acoustics and Content Understanding, Institute of Acoustics, Chinese Academy of Sciences, Beijing 100190, China; huangzhaoqiong@hccl.ioa.ac.cn (Z.H.); yanyonghong@hccl.ioa.ac.cn (Y.Y.); 2 University of Chinese Academy of Sciences, Beijing 100049, China; gzx@mail.ioa.ac.cn (Z.G.); whb@mail.ioa.ac.cn (H.W.); 3 State Key Laboratory of Acoustics, Institute of Acoustics, Chinese Academy of Sciences, Beijing 100190, China; 4 Xinjiang Key Laboratory of Minority Speech and Language Information Processing, Xinjiang Technical Institute of Physics and Chemistry, Chinese Academy of Sciences, Urumqi 830011, China

**Keywords:** multiple source localization, deep neural network, subarray beamforming, shallow water environment

## Abstract

Deep neural networks (DNNs) have been shown to be effective for single sound source localization in shallow water environments. However, multiple source localization is a more challenging task because of the interactions among multiple acoustic signals. This paper proposes a framework for multiple source localization on underwater horizontal arrays using deep neural networks. The two-stage DNNs are adopted to determine both the directions and ranges of multiple sources successively. A feed-forward neural network is trained for direction finding, while the long short term memory recurrent neural network is used for source ranging. Particularly, in the source ranging stage, we perform subarray beamforming to extract features of sources that are detected by the direction finding stage, because subarray beamforming can enhance the mixed signal to the desired direction while preserving the horizontal-longitudinal correlations of the acoustic field. In this way, a universal model trained in the single-source scenario can be applied to multi-source scenarios with arbitrary numbers of sources. Both simulations and experiments in a range-independent shallow water environment of SWellEx-96 Event S5 are given to demonstrate the effectiveness of the proposed method.

## 1. Introduction

Multiple source localization in an ocean waveguide is a challenging task because of the interactions among multiple acoustic signals. Several multiple source localization methods have been proposed for tracking underwater targets in past decades. Matched-field processing (MFP) is a classical approach for underwater source localization by correlating the modeled field and the experimental field [1,2,3]. The range and depth of source are given by the global maximum in the ambiguity surface generated by MFP.

However, the model based methods usually require the environmental parameters to model the acoustic model in advance. Difficulty in obtaining complete knowledge of the real environment may lead to incorrect or inaccurate localization results. To reduce the dependence on environmental information, recently, many data-driven techniques are introduced to source localization in ocean waveguides [4,5,6,7,8,9,10,11,12,13,14]. In previous works, researchers applied deep neural networks (DNNs) to source localization in shallow water environments and obtained promising results [7,8,9,10,11,12,13,14]. However, these studies usually focus on single-source localization. In real-world environments, there are usually multiple sources emerging. Therefore, it is significant to solve the multi-source localization problem in real environments. For a multiple source localization task, several variants of MFP have been proposed through modified Bartlett functions [15,16], maximum likelihood (ML) estimation [17,18], maximum a posteriori (MAP) processors [19], and so forth. Besides, compressive sensing (CS) [20,21,22] or sparse Bayesian learning (SBL) [23] have been combined with beamforming or MFP to estimate sources’ locations in multi-source scenarios. To our best knowledge, there are a few methods that apply DNNs to multiple source localization. In a multi-source scenario, sources tend to emerge in various directions. The directions of sources will be a valuable clue to discriminate multiple sources (the source direction is also represented by source azimuth angle). In this paper, we propose a DNN based method for multiple source localization on underwater horizontal arrays (UHAs).

To apply DNNs to a multiple source localization task, generally, there are two ideas in previous studies. The first idea is to train a single neural network that detects the locations of multiple sources using the mixed signals emitted from various location combinations directly [24,25,26,27,28]. However, training a single network from mixtures to estimate the locations of multiple sources is not an easy task, the reasons of which include—(1) It is hard to traverse all the combinations of source locations with different azimuth angles and ranges (it is supposed that the source location is determined by azimuth angle and range). To get an idea of how much training is required, we consider the two-source scenario for example. We start with training the network with $1^{\circ }$ separation of azimuth angles from $0^{\circ }$ to $359^{\circ }$ (e.g., ($0^{\circ },1^{\circ }$), ($1^{\circ },2^{\circ }$),…, ($359^{\circ },0^{\circ }$)). Next we repeat the same procedure with $2^{\circ }$ to $180^{\circ }$ separations. Assuming the azimuth angle is integer, the combinations of azimuth angle are $C_{360}^{2}$ for two-source scenario. Then we also take the range combinations into consideration, the possible training combinations will be enormous because of the exhaustive training; (2) if we do not separate the mixed signal in advance, the feature for learning is highly correlated with the source combination. Thus the estimation would fail if the test sources’ location combination is mismatched with the training set, and the application will be limited. For example, in the two-source scenario with test source one at [$125^{\circ }$, 1.2 km] and test source two at [$220^{\circ }$, 2.5 km], if this combination does not exist in the training set, the single network (trained for two-source scenario) may fail to give an accurate estimation. Therefore, training the network suitable for various scenarios by mixtures directly is not an optimal scheme.

The second idea tries to simplify the multi-source localization task to single-source localization task. The most popular methods are based on the sparsity assumption on sound source signal [29,30]. Although simultaneous sources overlap in time, if the signal (e.g., speech signal), conforms to be sparsely distributed in the time-frequency (TF) domain, multiple sources will have different distributions in the frequency domain. Hence, this allows training using single-source data and the DNN-based single source localization methods can be conducted on each TF bin. Then, a fusion process is leveraged to integrate the localization results on all TF bins into the spatial information, such as the direction-of-arrivals (DOAs) and the number of multiple sources. However, the underwater sources usually cannot satisfy the sparsity assumption, so this idea is not suitable for our work.

To circumvent these problems, a two-stage DNN based method is proposed to determine both the azimuth angles and ranges of multiple sources successively, which includes a feed-forward neural network (FNN) for direction finding and a long short term memory recurrent neural network [31] (LSTM-RNN) for source ranging. Basically, there are three originalities of our proposed framework. First, in a feature extraction module, we design a subarray beamforming [32] based feature extractor to separate multiple sources at the level of feature, so that the multi-source localization can be simplified to the single-source localization. Consider the horizontal-longitudinal correlations of the low-frequency acoustic field [33], the UHA is divided into several subarrays and the conventional beamforming (CBF) [34] is conducted on each subarray. The spatial correlation matrix (SCM) of the beamformed signals at all subarrays is taken as the feature. Second, since different sources are discriminated by the features, the multiple sources’ ranges can be respectively estimated by the DNN model trained in the single-source scenario. Besides, the LSTN-RNN is adopted to take full advantage of long-term temporal contextual information for the current estimation. Third, an FNN-based direction finding method is presented. A FNN model with a back propagation (BP) algorithm [35] is trained to find the possible directions of sources and determine source number. Then the features of multiple sources can be extracted based on the direction candidates. With subarray beamforming and two-stage DNNs, the need to include multi-source data for training is avoided and the model trained by single-source data can be applied to the multi-source scenarios with arbitrary numbers of sources. In particular, we can localize sources that even overlap fully in the frequency domain.

The rest of the paper is organized as follows. Section 2 formulates the signal model. Section 3 describes the proposed method and each module in detail. Section 4 and Section 5 give various simulations and experiments for evaluation. Finally, Section 6 concludes this work.

## 2. Signal Model

Consider *D* broadband sound sources impinge on an array of *K* hydrophones in a far-field scenario, the signal at frequency $f_{i}$ received by the hydrophones is described as

(1) $$Y\left(f_{i}\right)=\sum_{d=1}^{D}S_{d}\left(f_{i}\right)A\left(\theta_{d},f_{i}\right)+N\left(f_{i}\right),\;\;i\in \left\{1,\cdots ,F\right\},$$

where $S_{d}\left(f_{i}\right)$ denotes the *d*th signal, $A(\theta_{d},f_{i})$ denotes the K×1 steering vector corresponding to the *d*th source, $\theta_{d}$ denotes the DOA of the *d*th signal, $N(f_{i})$ denotes the noise at the hydrophones, *i* denotes the frequency index, and *F* denotes the number of frequency bins. Denote

(2) $$\begin{array}{cc} H(\theta_{d},f_{i}) & =A\left(\theta_{d},f_{i}\right)/||A\left(\theta_{d},f_{i}\right){\left|\right|}_{2},\\ x_{d}\left(f_{i}\right) & =S_{d}\left(f_{i}\right)||A\left(\theta_{d},f_{i}\right){\left|\right|}_{2}, \end{array}$$

Equation (1) can be rewritten using the matrix notation as

(3) $$Y\left(f_{i}\right)=H\left(f_{i}\right)X\left(f_{i}\right)+N\left(f_{i}\right),$$

where $H\left(f_{i}\right)=\left[H\left(\theta_{1},f_{i}\right),\cdots ,H\left(\theta_{D},f_{i}\right)\right]$ is a K×D steering matrix defining all the potential positions, $H^{H}\left(\theta_{d},f_{i}\right)H\left(\theta_{d},f_{i}\right)=1$, $X\left(f_{i}\right)={\left[x_{1}\left(f_{i}\right),\cdots ,x_{D}\left(f_{i}\right)\right]}^{T}$ is a D×1 dimensional vector denoting the signal, ${\left(\cdot \right)}^{H}$ denotes the Hermitian transpose, and ${\left(\cdot \right)}^{T}$ denotes the transpose.

The DOA $\theta_{d}$ is represented by the azimuth angle $\alpha_{d}$ and the grazing angle $\beta_{d}$,

(4) $$\theta_{d}={\left[\cos\alpha_{d}\cos\beta_{d},\sin\alpha_{d}\cos\beta_{d},\sin\beta_{d}\right]}^{T}.$$

**Figure 1:**
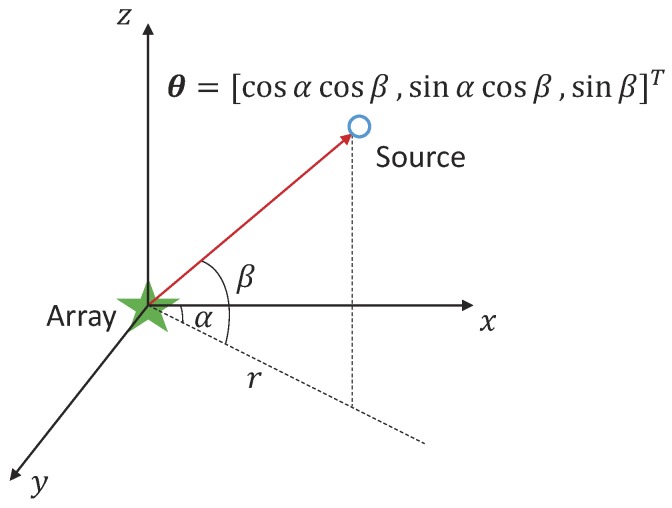
Geometrical relationship of direction of arrival (DOA) (θ) and the azimuth angle (α) and the grazing angle (β). A horizontal array is deployed at the xy plane. The horizontal distance between source and array is *r* km.

The geometrical relationship of the DOA (θ) and the azimuth angle (α) and the grazing angle (β) is shown in Figure 1. For horizontal array, the grazing angle of propagation is small in the far-field scenario ($\beta <20^{\circ }$) [36], that is, cosβ≈1. Therefore, the steering vector depends mainly on the azimuth angle α. For simplicity, $\theta_{d}$ is approximated to ${\left[\cos\alpha_{d},\sin\alpha_{d},0\right]}^{T}$ in the following process.

## 3. Proposed Method

**Figure 2:**
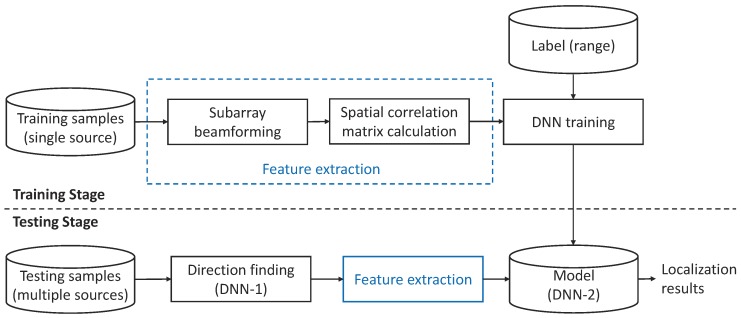
Block diagram of the proposed method.

The block diagram of the proposed method is shown in Figure 2. In the training stage, the features are extracted from the single source signal radiated from different locations by performing subarray beamforming and calculating the SCM of the beamformed signals at all subarrays. Then DNN-2 is trained to model the regression relationship between the extracted feature and the source range. In the testing stage, the azimuth angles of sources are firstly estimated by DNN-1. The features of sources are extracted based on all azimuth angle candidates at subarrays. Finally, the range of each source is inferred by feeding the feature associated with each source to DNN-2.

### 3.1. Direction Finding

**Figure 3:**
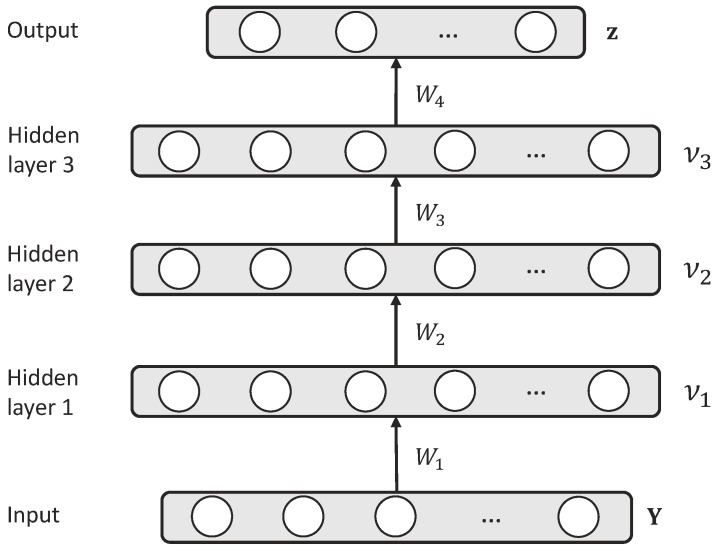
The architecture of FNN/DNN-1.

Rstogi et al. proposed using the hopfield network [37] in direction finding [38]. The basic idea is to use a neural network to find the best possible choice of directions present in the received signal through minimizing a quadratic cost function. Compared to the conventional neural network, DNN with a BP algorithm has a stronger capability for finding the good solutions to a difficult optimization problem. However, there are few methods that apply DNNs to direction finding in the ocean environments. In this paper, we attempt to get desirable results of sources’ directions using a FNN. The configuration of FNN (i.e., DNN-1 in Figure 2) is shown in Figure 3, where the projection from the input vector $\nu_{\iota }$ at the ιth layer to the output vector $\nu_{\iota +1}$ at the (ι+1)th layer is represented as

(5) $$\nu_{\iota +1}=W_{\iota }\nu_{\iota }+b_{\iota },$$

where $W_{\iota }$ and $b_{\iota }$ denote the weight and bias matrix from the ιth layer to the (ι+1)th layer. The feature of DNN-1 is the FFT coefficients of the observed signal Y. The real and imaginary part of FFT coefficients are concatenated as the input of DNN-1. Denote $H\left(\theta_{d},f_{i}\right)={\left[1,\;e^{j2\pi f_{i}\tau_{2}},\;e^{j2\pi f_{i}\tau_{3}},\cdots ,e^{j2\pi f_{i}\tau_{K}}\right]}^{T}$ ($\tau_{k}$ is the time delay between the *k*th hydrophone and the first hydrophone), which is the steering vector of the *d*th source, the cost function for the broadband case can be expressed as

(6) $$\begin{array}{c} \Lambda =\frac{1}{L\times F}\sum_{l=1}^{L}\sum_{i=1}^{F}||Y_{l}\left(f_{i}\right)-\left[\Gamma_{f,1}Y_{l}\left(f_{i}\right)\;\cdots \;\Gamma_{f,P}Y_{l}\left(f_{i}\right)\right]z|{|}^{2}, \end{array}$$

where $\Gamma_{f,p}=H\left(\theta_{d},f_{i}\right)\left[H^{H}\left(\theta_{d},f_{i}\right)H\left(\theta_{d},f_{i}\right)\right]H^{H}\left(\theta_{d},f_{i}\right)$, *L* denotes the the snapshot number and $z={\left[z_{1},z_{2},\cdots ,z_{P}\right]}^{T}$$\left(z_{p}\in \left[0,1\right]\right)$ is the output vector of the neural network. $\Gamma_{f,1}Y_{l}\left(f_{i}\right)$ is the K×1 dimensional vector of the observed signal projected onto the steering vector $H(\theta_{d},f_{i})$. The cost function will be minimized by the best linear combination of the steering vectors, when convergence, the extremums in vector z indicate the possible sources.

Each significant peak of vector z is identified as a sound source, the probability of which is greater than the threshold,

(7) $$\delta =O_{avg}+\eta \left(O_{max}-O_{avg}\right),$$

where $O_{avg}$ and $O_{max}$ denotes the average and maximum of the smoothed probabilities, and the coefficient η (0<η<1) is set by experiment.

Note that only in the testing stage is the FNN using BP algorithm trained to find the directions that sound sources may emerge. For each direction candidate, we extract the corresponding features, then the sources’ ranges are estimated by feeding the features into DNN-2 (i.e., LSTM-RNN).

### 3.2. Source Ranging

To avoid the exhaustive training, we aim to train a general and flexible model that is suitable for situations with different source numbers. Thus, how to design an effective feature, which can be used for various scenarios, is a critical problem. For DNN analysis, the more similar the test set is to the training set, the better the testing result will be. However, in our task, the training set is composed by the single-source signals at different locations while only the mixture is available when testing. It is vital to extract a feature that can represent each single source information from the mixture, so that the test signal (or feature) can be matched with the training signals. Beamforming, which can enhance the signal from the desired direction while attenuating others, is ideal to extract the individual signal component from the mixture. Nevertheless, if we perform beamforming using all sensors, the horizontal-longitudinal correlations of the acoustic field, which include the spatial information of source, will lost in the enhanced signal. Therefore, we introduce subarray beamforming to extract the individual source component, meanwhile preserving the horizontal-longitudinal correlations. The SCM of the enhanced signals at all subarrays is used as the feature.

#### 3.2.1. Feature Extraction

**Figure 4:**
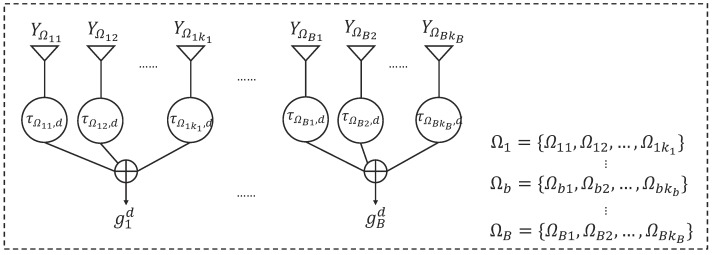
Block diagram of subarray beamforming.

Beamforming algorithms can be used to track those interested sources and null out the other sources as interference by controlling the beampattern of an array. The simplest beamforming technique is adopted in our framework, which refers to the delay-and-sum beamforming. It delays the multi-channel signals so that all versions of the source signal are time-aligned before they are summed. To preserve the horizontal-longitudinal correlations of the low-frequency acoustic field, this CBF is conducted on each subarray. The hydrophone array is divided to *B* subarrays, $\left\{\Omega_{1},\cdots ,\Omega_{B}\right\}$, then the signal enhanced to the *d*th direction at the *b*th subarray is obtained by applying CBF to the signals received by the hydrophones in the *b*th subarray,

(8) $$\begin{array}{cc} g_{b}^{d}\left(f_{i}\right)= & \sum_{k\in \Omega_{b}}Y_{k}\left(f_{i}\right)e^{-j2\pi f_{i}\tau_{k,d}},\\ \tau_{k,d}= & \ell_{k}\gamma_{k}^{T}\theta_{d}/c, \end{array}$$

where $\tau_{k,d}$ denotes the *d*th time delay of the *k*th hydrophone corresponding to the first hydrophone at the *b*th subarray (the first hydrophone is chosen as the reference), $\ell_{k}$ and $\gamma_{k}^{T}$ denote the distance and the unit directional vector between the *k*th hydrophone and the reference hydrophone, $\Omega_{b}$ denotes the hydrophone index set of the *b*th subarray, *c* denotes the sound speed and $j=\sqrt{-1}$ denotes the imaginary unit. The enhanced signals of the *d*th source at frequency $f_{i}$ obtained by all subarrays are given by $G_{d}\left(f_{i}\right)={\left[g_{1}^{d}\left(f_{i}\right),\cdots ,g_{B}^{d}\left(f_{i}\right)\right]}^{T}$. The block diagram of subarray beamforming is shown in Figure 4.

The SCM of the signals enhanced to each source direction is used as the feature, because it contains sufficient information about the individual signal. The SCM of the *d*th source is calculated by

(9) $$R_{d}\left(f_{i}\right)=E\left[{\tilde{G}}_{d}\left(f_{i}\right){\tilde{G}}_{d}^{H}\left(f_{i}\right)\right],$$

where ${\tilde{G}}_{d}\left(f_{i}\right)=G_{d}\left(f_{i}\right)/\left|\right|G_{d}\left(f_{i}\right)\left|\right|$. The real and imaginary part of the upper triangular matrix of the SCM is concatenated as a B×(B+1) dimensional vector denoted by $u_{d}$, which is used as the input feature of the neural network.

#### 3.2.2. DNN Analysis with LSTM-RNN

DNN [39] is a data-driven technique that learns the potential patterns from the original acoustic data directly. Due to the movement of the source, we take source localization to be a regression task. In the regression problem, the target output r∈(0,∞) is a continuous range variable. For the source localization task, current range of a source is considered to be related to its adjacent locations. However, FNN, or time delay neural network [40] (TDNN), can provide only limited temporal modeling by splicing fixed frames of features in the input or hidden layers. By contrast, RNNs contain cycles that feed the network activations from a previous time step as inputs to the network to influence predictions at the current time step, so the more sufficient long-term temporal contextual information can be used. In particular, LSTM architecture [31] overcomes the gradients vanishing and exploding existing in traditional RNNs by introducing some special units called memory blocks. Therefore, we adopt LSTM-RNN to model the mapping between the feature and source range in our framework.

**Figure 5:**
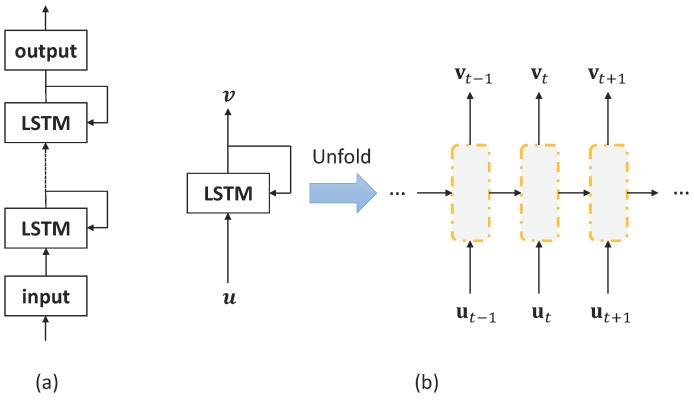
The configuration of LSTM-RNN. (**a**) The deep LSTM-RNN; (**b**) The configuration of LSTM memory blocks that unfolded across time.

**Figure 6:**
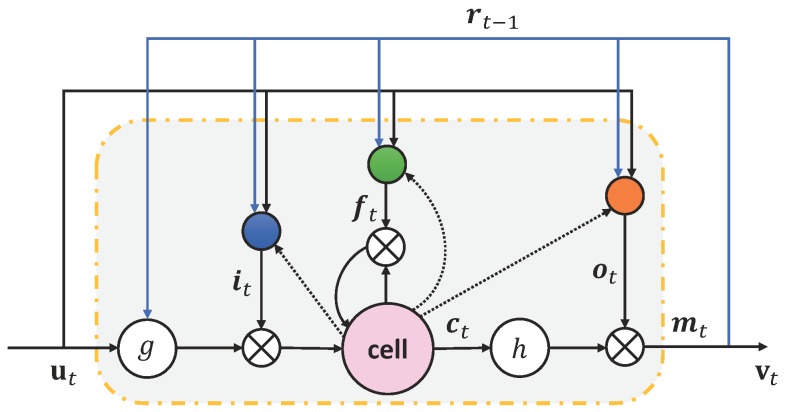
The configuration of LSTM memory block.

The deep LSTM-RNN is shown in Figure 5a, and the configuration of LSTM memory blocks is shown in Figure 5b, where the input and output vectors are denoted as $u=(u_{1},\cdots ,u_{T})$ and $v=(v_{1},\cdots ,v_{T})$. The configuration of LSTM memory blocks that unfolded across time (the yellow dashed box in Figure 5a) is shown in Figure 6. The memory block contains several self-parameterized controlling gates, i.e., input gate, output gate, and forget gate, to control the flow of information. The input gate controls the flow of input activations into the memory cell. The output gate controls the output flow of cell activations into the rest of the network. Finally, the forget gate is added to forget or reset the cell’s memory adaptively.

The associated computations that map the input vector to the output vector are given as follows:

(10) $$\begin{array}{cc} i_{t} & =\sigma (W_{iu}u_{t}+W_{im}m_{t-1}+W_{ic}c_{t-1}+b_{i}) \end{array}$$

(11) $$\begin{array}{cc} f_{t} & =\sigma (W_{fu}u_{t}+W_{fm}m_{t-1}+W_{fc}c_{t-1}+b_{f}) \end{array}$$

(12) $$\begin{array}{cc} c_{t} & =f_{t}⊙c_{t-1}+i_{t}⊙g\left(W_{cu}u_{t}+W_{cm}m_{t-1}+b_{c}\right) \end{array}$$

(13) $$\begin{array}{cc} o_{t} & =\sigma (W_{ou}u_{t}+W_{om}m_{t-1}+W_{oc}c_{t}+b_{o}) \end{array}$$

(14) $$\begin{array}{cc} m_{t} & =o_{t}⊙h\left(c_{t}\right) \end{array}$$

(15) $$\begin{array}{cc} v_{t} & =m_{t} \end{array}$$

where i, f, o, c, m denote the input gate, forget gate, output gate, cell activation, and cell output activation vectors respectively, W terms denote the weight matrices, in which $W_{ic}$, $W_{fc}$, $W_{oc}$ are diagonal weight matrices for peephole connections (the dotted lines from cell to gates in Figure 6), b denotes the bias matrices, σ denotes the sigmoid activation function, ⊙ denotes the element-wise product, *g* and *h* are the cell input and cell output activation functions that are tanh in this paper.

The cost function is defined as the mean square error (MSE) between the estimated source range $r_{q}$ and the reference source range ${\hat{r}}_{q}$, given by

(16) $$E=\frac{1}{Q}\sum_{q=1}^{Q}{\left(r_{q}-{\hat{r}}_{q}\right)}^{2},$$

where *Q* denotes the sample number. We use the truncated back propagation through time (BPTT) learning algorithm [41] to update the parameters.

#### 3.2.3. Data Augmentation

In our framework, the two-stage DNNs are used to determine both the azimuth angles and ranges of multiple sources. In source ranging stage, we need azimuth angles that estimated by DNN-1 to perform feature extraction for DNN-2. The accuracy of estimated source range by DNN-2 is not only determined by DNN-2, but also the feature extracted based on the estimated results of DNN-1. Therefore, if the azimuth angles are inaccurately estimated by DNN-1, the features generated based on the deviant azimuth angles may lead to differences from the correct features (i.e., the result of subarray beamforming using the estimated azimuth angle α is different from that using the true azimuth angle $\hat{\alpha }$). Therefore, the error introduced by direction finding may cause the inaccurate estimation of source range. To reduce the negative effect of direction finding on source ranging and improve the generalization ability of DNN-2, we introduce some disturbances in feature extraction and the disturbed features are merged to the training set in the training stage of DNN-2. This strategy is called data augmentation [42,43,44] (which is widely used in speech recognition or speech enhancement). The original data, denoted as Φ, are disturbed during feature extraction stage to obtain the augmented features, denoted as Ψ. Explicitly, for each sample in Φ, we obtained the augmented feature $u_{\zeta }^{\kappa }$ (where the superscript κ denotes the sample index in Φ) by introducing an offset angle $\alpha_{\zeta }$ to the true azimuth angle $\hat{\alpha }$. The augmented beamformed signal calculated by the disturbed azimuth angle $\alpha^{\prime }=\hat{\alpha }+\alpha_{\zeta }$ is obtained by modifying Equation (8) as

(17) $$\begin{array}{cc} g_{b}^{\prime }\left(f_{i}\right)= & \sum_{k\in \Omega }Y_{k}\left(f_{i}\right)e^{-j2\pi f_{i}\tau_{k}^{\prime }},\\ \tau_{k}^{\prime }= & \ell_{k}\gamma_{k}^{T}\theta^{\prime }/c, \end{array}$$

where $\theta^{\prime }={\left[\cos\alpha^{\prime },\sin\alpha^{\prime },0\right]}^{T}$. The augmented feature $u_{\zeta }^{\kappa }$ is obtained by calculating the SCM of the augmented signals at all subarrays, $G^{\prime }\left(f_{i}\right)={\left[g_{1}^{\prime }\left(f_{i}\right),\cdots ,g_{B}^{\prime }\left(f_{i}\right)\right]}^{T}$. The data augmentation process is detailed as Table 1 (Algorithm 1), where ϑ limits the range of angle offset and $\vartheta_{o}$ is the step size.

## 4. Simulations

### 4.1. Acoustic Environmental Model

**Figure 7:**
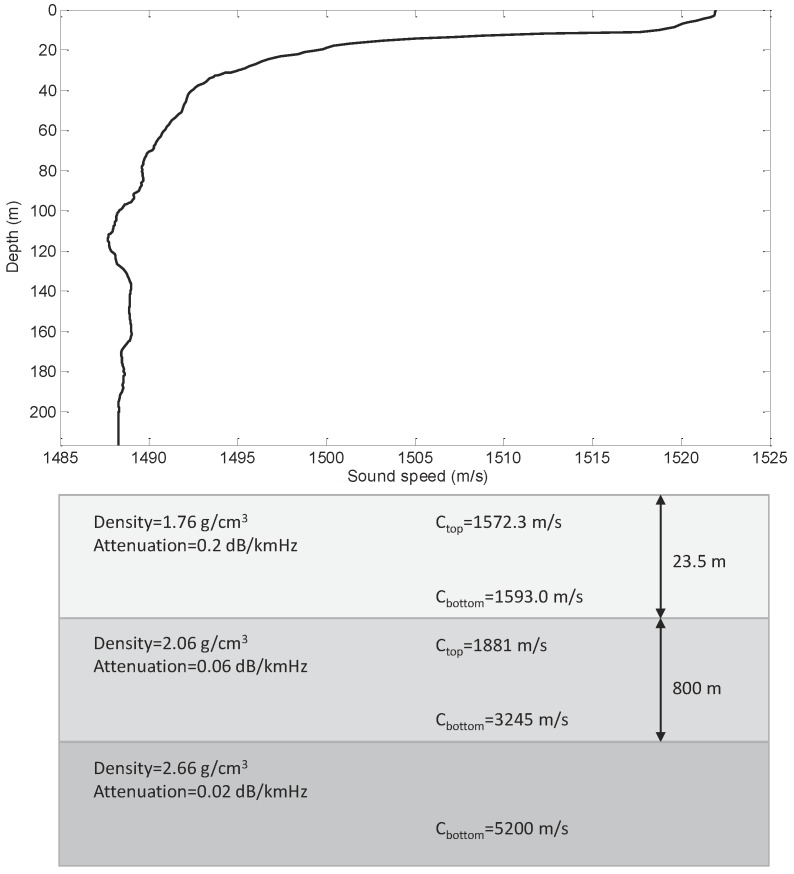
Waveguide with sound speed profile and geoacoustic parameters for range-independent SWellEx-96 Event S5.

To investigate the performance of the proposed method, we simulated the relatively range independent SWellEx-96 Event S5 [45] environment. The sound speed profile (SSP) and geoacoustic parameters for SWellEx-96 Event S5 are shown in Figure 7. The seafloor is composed first of a 23.5 m thick sediment layer with a density of 1.76 g/cm${}^{3}$ and an attenuation of 0.2 dB/kmHz. The top and bottom sound speeds are 1572.368 m/s and 1593.016 m/s. Below the sediment layer is an 800 m thick mudstone layer with a density of 2.06 g/cm${}^{3}$ and an attenuation of 0.06 dB/kmHz. The top and bottom sound speeds of the mudstone layer are 1881 m/s and 3245 m/s. The geoacoustic model is completed by a halfspace with a density of 2.66 g/cm${}^{3}$, an attenuation of 0.02 dB/kmHz, and a compressional sound speed of 5200 m/s.

### 4.2. Data Description

**Figure 8:**
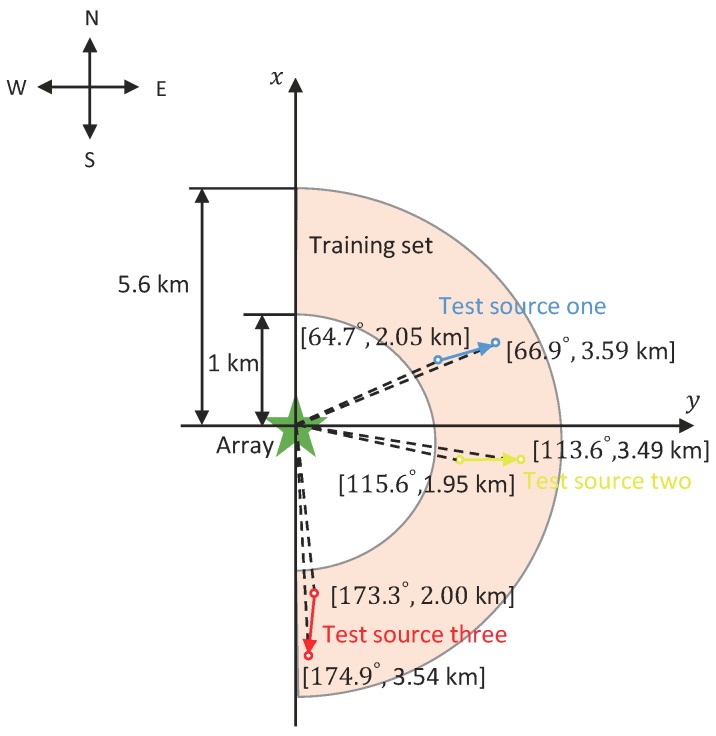
The map of source movement and the location of the hydrophone array in the simulation. The semi-annular orange region covers the ranges of training sources’ motions. The training data included sources with azimuth angles from $0^{\circ }$ to $180^{\circ }$ with $5^{\circ }$ intervals (the course equals to azimuth angle). In each azimuth angle, the source was ranging from 1.0 to 5.6 km at a speed of 5 knots (2.5 m/s). The blue, yellow, and red lines were the trajectories of test source one, two, and three. The array includes two topologies, including HCA and HLA. The HCA was 50-element with a 250 m radius, where the hydrophones were uniformly distributed. The HLA was 27-element, the layout of which is the same as the HLA North of SWellEx-96 Event S5.

In the simulation, the bandwidth of signal was [50, 210] Hz and the sampling rate was 3276.8 Hz. The hydrophone array was deployed at a 213 m depth of water. We investigated two topologies of UHAs, including a horizontal circular array (HCA) and a horizontal line array (HLA) (note that our method is suitable for UHA with arbitrary topologies). The HCA was 50-element with a 250 m radius, where the hydrophones were uniformly distributed. The HLA was 27-element, the layout of which was the same as that of the HLA North of SWellEx-96 Event S5 (the details can refer to the web page http://swellex96.ucsd.edu/hla_north.htm). In fact, the line array was not strictly linear but had a certain degree of curvature. The map of source movement and the location of the hydrophone array are depicted in Figure 8. The training data included sources with azimuth angles from $0^{\circ }$ to $180^{\circ }$ with $5^{\circ }$ intervals (the course equals to azimuth angle). In each azimuth angle, the source ranged from 1.0 to 5.6 km at a speed of 5 knots (2.5 m/s). The source depth was fixed to 54 m. When testing, every testing segmentation contained ten minutes (including 960 samples) and the two-source scenario included source one from [$64.7^{\circ }$, 2.05 km] to [$66.9^{\circ }$, 3.59 km], and source two from [$115.6^{\circ }$, 1.95 km] to [$113.6^{\circ }$, 3.49 km]. The three-source scenario included source one from [$64.7^{\circ }$, 2.05 km] to [$66.9^{\circ }$, 3.59 km], source two from [$115.6^{\circ }$, 1.95 km] to [$113.6^{\circ }$, 3.49 km], and source three from [$173.3^{\circ }$, 2.00 km] to [$174.9^{\circ }$, 3.54 km]. The training data and testing data were mutually different.

The signal was transformed to the frequency domain by operating fast Fourier transformation (FFT) (Hanning windowed). The frame length was 1.25 s with 50% overlap. The bandwidth for processing was set to [100,200] Hz (with 5 Hz increment, totally 21 frequency bins). For HCA, the 50 hydrophones were divided into five subarrays uniformly, that is, $\Omega_{1}=\left\{1,\cdots ,10\right\}$, $\Omega_{2}=\left\{11,\cdots ,20\right\}$, $\Omega_{3}=\left\{21,\cdots ,30\right\}$, $\Omega_{4}=\left\{31,\cdots ,40\right\}$, and $\Omega_{5}=\left\{41,\cdots ,50\right\}$. For HLA, the 27 hydrophones were divided into four subarrays, the hydrophone indexes of subarrays were $\Omega_{1}=\left\{1,\cdots ,7\right\}$, $\Omega_{2}=\left\{8,\cdots ,14\right\}$, $\Omega_{3}=\left\{15,\cdots ,21\right\}$, and $\Omega_{4}=\left\{22,\cdots ,27\right\}$. Twenty snapshots were used to calculate the SCM. Data augmentation was performed using $\vartheta =7^{\circ }$ and $\vartheta_{o}=0.5^{\circ }$, generating about $3.1\times 10^{6}$ training samples.

### 4.3. The Configuration of DNNs

In direction finding, the configuration of FNN was 5 layers (one input layer + three hidden layers + one output layer) with 128 hidden nodes. The rectified linear units [46] (ReLU), f(x)=max(0,x), was used as the activation function. The initial learning rate was 0.001 and the batch size was 6. The input of FNN was the FFT coefficients of each frame, so the input dimension of FNN were 1134 (27×2×21, real and imaginary parts were concatenated) for HLA and 2100 (50×2×21) for HCA.

In source ranging, the LSTM-RNN was three layers with 896 nodes. The activation function was ReLU. The initial learning rate was 0.001 and the batch size was 512. The input dimension of LSTM-RNN were 420 (4×5×21) for HLA and 630 (5×6×21) for HCA.

It should be mentioned that all parameters (e.g., hidden nodes, hidden layers, learning rate, and batch size) of FNN or LSTM-RNN were chosen based on experiments. The tensorflow [47] toolkit was taken for FNN and LSTM-RNN training. Adam [48] was utilized for optimization.

### 4.4. Metrics

#### 4.4.1. Direction Finding

For direction finding, the detected sources were classified into two categories, namely the correctly detected sources and the incorrectly detected sources. The detection was considered to be correct if the estimated azimuth angle deviated no more than $7^{\circ }$ from the real azimuth angle of any source. The incorrectly detected sources consisted of the imaginary sources (detected but non-existing sources) and the inaccurately detected sources. The detection correctness was mainly evaluated in terms of the positive detection rate (PDR) (i.e., the ratio of the number of correctly detected sources to the total number of sources) and the false detection rate (FDR) (i.e., the ratio of the number of incorrectly detected sources to the total number of sources). The receiver operating performance characteristics (ROC) curve gave a complete description of the relationship between PDR and FDR with the change of threshold η (0 to 0.95 with 0.05 steps). Define

(18) $$\eta_{o}=\min_{\eta }\left|1-\mathrm{PDR}\left(\eta \right)+\mathrm{FDR}\left(\eta \right)\right|,$$

the mean absolute error (MAE) between the true azimuth angles and the estimated azimuth angles of correctly detected sources when $\eta =\eta_{o}$ was combined with ROC curve to evaluate the performance in direction finding stage. The MAE between the true azimuth angles ($\hat{\alpha }$) and the estimated azimuth angles (α) is defined as

(19) $${\mathrm{MAE}}_{\alpha }=\frac{1}{\Xi }\sum_{\xi =1}^{\Xi }\min_{d\in \{1,\cdots ,D\}}F\left(\alpha_{\xi }-{\hat{\alpha }}_{\xi ,d}\right),$$

where F(α) is denoted as

(20) $$F\left(\alpha \right)=\min_{n}|\alpha +360^{\circ }\times n|,$$

where *n* is an integer denoting the number of azimuth period, $F\left(\alpha \right)\in \left[0,180^{\circ }\right]$, and Ξ denotes the number of estimation results and ξ is the sample index.

#### 4.4.2. Source Ranging

The objective evaluation metrics used for source ranging were the MAE and the mean relative error (MRE) between the estimated ranges (*r*) and the true ranges ($\hat{r}$),

(21) $${\mathrm{MAE}}_{r}=\frac{1}{\Xi }\sum_{\xi =1}^{\Xi }\left|r_{\xi }-{\hat{r}}_{\xi }\right|,$$

(22) $${\mathrm{MRE}}_{r}=\frac{1}{\Xi }\sum_{\xi =1}^{\Xi }\frac{|r_{\xi }-{\hat{r}}_{\xi }|}{{\hat{r}}_{\xi }}\times 100\%.$$

### 4.5. Simulation Results

**Figure 9:**
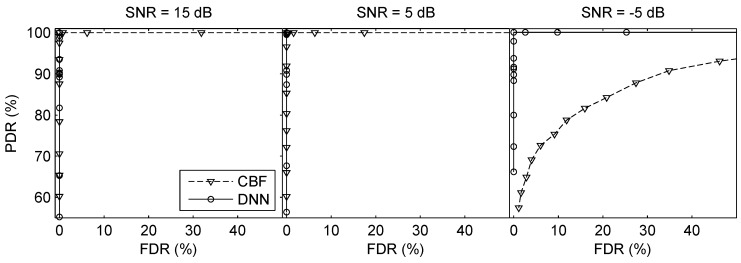
ROC curves of direction finding on HCA under different SNRs in the two-source scenarios.

**Figure 10:**
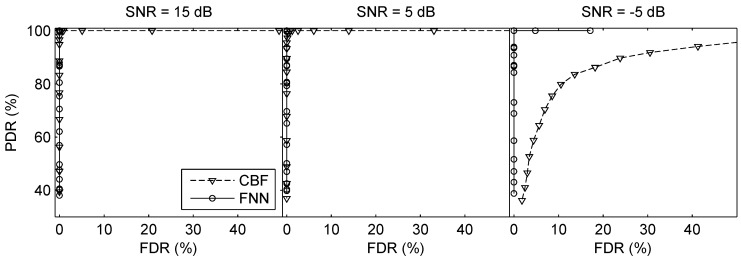
ROC curves of direction finding on HCA under different SNRs in the three-source scenarios.

The first simulation was conducted to investigate the performance of the proposed method under different signal-to-noise ratios (SNRs). White noise was added to the simulated signals, resulting in SNRs of 15, 5, and −5 dB. The SNR [49] reported here was defined as the SNR (at 210 Hz) at a single hydrophone when the source range was 1 km (SNR would decrease with source range increasing). Both source level (SL) and noise level (NL) were attenuated by −6 dB/Oct. The CBF [34] was chosen as the competing algorithms in direction finding. Twenty snapshots were used to calculate beamformer power of CBF. For the sake of fairness, the posterior probability of FNN was averaged over every twenty frames. The results of the two-source scenarios and three-source scenarios on HCA are summarized in Table 2. The ROC curves of two-source scenario and three-source scenario are plotted in Figure 9 and Figure 10 (The SNR shown here is the SNR of the received signal for each source, and the SL of each source is assumed to be equal). It should be mentioned that, the number of points seen on the figures may be less than the number of points actually sampled, because (1) there are some η correspond to the same PDR and FDR and they are overlapped in the figures; (2) there are some points of CBF go out of scope because of the large FDR when η is small. From the ROC curves, although the performance degrades with the lower SNR, the FNN and CBF can detect sources effectively in general. Superficially, the three methods can give a high PDR with a low FDR by setting an appropriate threshold; however, the values of $\eta_{o}$ of CBF are larger than FNN significantly. The smaller $\eta_{o}$ implies the stronger ability of suppressing the interference. Thus, there are little phantom peaks of FNN than CBF, which is a good indication of its better capability of suppressing interference. When SNR decreases to −5 dB, the FDR of CBF rises and PDR decreases, which reveals the proposed method is more robust than CBF under a lower SNR. Furthermore, the estimation errors of FNN are smaller than CBF in all conditions as shown in Table 2.

For source ranging, we compared the performance of LSTM-RNN with FNN. The FNN was five layers with three hidden layers and 896 hidden nodes. From Table 2, the LSTM-RNN outperforms FNN, which demonstrates the superiority of LSTM-RNN in modeling the long-term temporal information. In addition, we may notice that the locations of the test sources may not exist in the training set. However, the proposed method can still give reliable estimates to sources’ ranges, which reveals that the proposed method can localize the sources as long as the test source locations are in the region of the training set.

We also evaluated the performance on HLA under different SNRs. The results are summarized in Table 3. We can find that the proposed method also exhibits a good performance on direction finding and source ranging on HLA. Comparing Table 2 and Table 3, basically, the performance of the proposed method is similar to different array topologies. Whereas the ${\mathrm{MAE}}_{\alpha }$ of HLA is larger than HCA, the reason of which considers the angular resolution of HCA is constant with the change of azimuth angles while it varies for HLA. The experimental results indicate that the proposed method can be applied to the UHA with arbitrary topologies. For simplicity, the following simulations were all conducted on HCA.

The second simulation evaluated the performance with or without data augmentation in the two-source scenario. The SNR was set to 5 dB and the neural network was LSTM-RNN. The ${\mathrm{MAE}}_{r}$ and ${\mathrm{MRE}}_{r}$ without data augmentation are 0.56 km and 20.9%. From Table 3, with data augmentation, the ${\mathrm{MAE}}_{r}$ and ${\mathrm{MRE}}_{r}$ drop to 0.09 km and 3.4% respectively. The results demonstrate that data augmentation can improve the generalization ability of DNN model.

**Figure 11:**
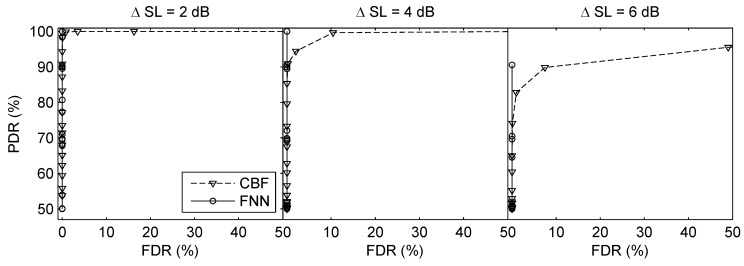
ROC curves of direction finding when the SLs of two testing sources are different in the two-source scenarios. The SNR of the dominant source was 5 dB. $\Delta \mathrm{SL}={\mathrm{SL}}_{1}-{\mathrm{SL}}_{2}$ (dB) (${\mathrm{SL}}_{1}$ corresponds to the dominant source and ${\mathrm{SL}}_{2}$ corresponds to the weak source).

The third simulation was made to investigate the performance of the proposed method when the SLs of two testing sources were different, where the source with the higher SL referred to the dominant source. The SNR of the dominant source was 5 dB. Define $\Delta \mathrm{SL}={\mathrm{SL}}_{1}-{\mathrm{SL}}_{2}$ (dB) (${\mathrm{SL}}_{1}$ corresponds to the dominant source and ${\mathrm{SL}}_{2}$ corresponds to the weak source), Figure 11 compares the ROC curves of CBF and FNN when ΔSL=2,4,6 dB. Both methods can give high PDR with low FDR when two SLs are comparable. Nevertheless, the false detections of CBF rise faster than FNN when the difference between the two SLs increases. In addition, the ${\mathrm{MAE}}_{r}$ and ${\mathrm{MRE}}_{r}$ of source ranging are summarized in Table 4. With ΔSL increasing, the estimation error increases because the weak source is masked by the presence of the dominant source, which leads to the larger error of the weak source.

**Figure 12:**
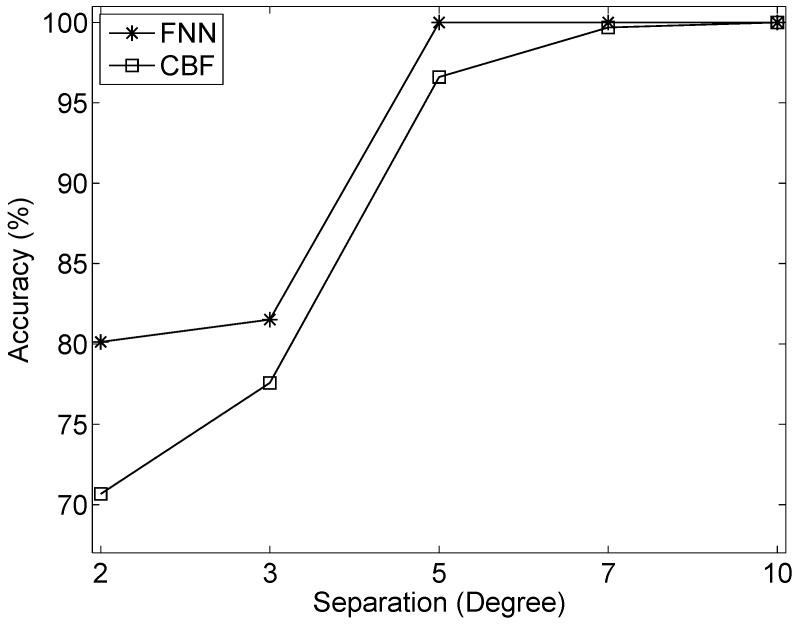
Detection accuracies of FNN and CBF. The detection is deemed to be correct only when the source number and the azimuths of two sources are estimated correctly. The accuracy is defined as the ratio of the number of accurate detections and the number of test samples.

The last experiment investigated the spatial resolution of the proposed method. The separations of two sources were set to $2^{\circ }$, $3^{\circ }$, $5^{\circ }$, $7^{\circ }$, and $10^{\circ }$. Here, the azimuth of each source was fixed, while the range of each source was from 1 km to 2.5 km. The SNR was set to 5 dB. The detection accuracies of FNN and CBF in direction finding are shown in Figure 12. Here, only when the source number and the azimuth angles of two sources are estimated correctly is the detection deemed to be correct. The accuracy is defined as the ratio of the number of accurate detections and the number of test samples. From Figure 12, generally, FNN and CBF can discriminate two widely separated sources, and the accuracy of FNN outperforms CBF. When the separation of two sources becomes smaller, FNN presents its superiority in discriminating two closely separated sources. We evaluated the performance of source ranging using LSTM-RNN. The results of source ranging are summarized in Table 5, where the ${\mathrm{MAE}}_{r}$ and ${\mathrm{MRE}}_{r}$ are calculated using the test samples with the accurate estimated azimuth angles. The results show that the separations have little influence on source ranging if the azimuth angles are estimated accurately. Note that the ${\mathrm{MAE}}_{r}$ and ${\mathrm{MRE}}_{r}$ are slightly smaller than those shown in Table 2, because the range of testing sources here are nearer than those in the first simulation.

## 5. Experiments

### 5.1. Experimental Database

**Figure 13:**
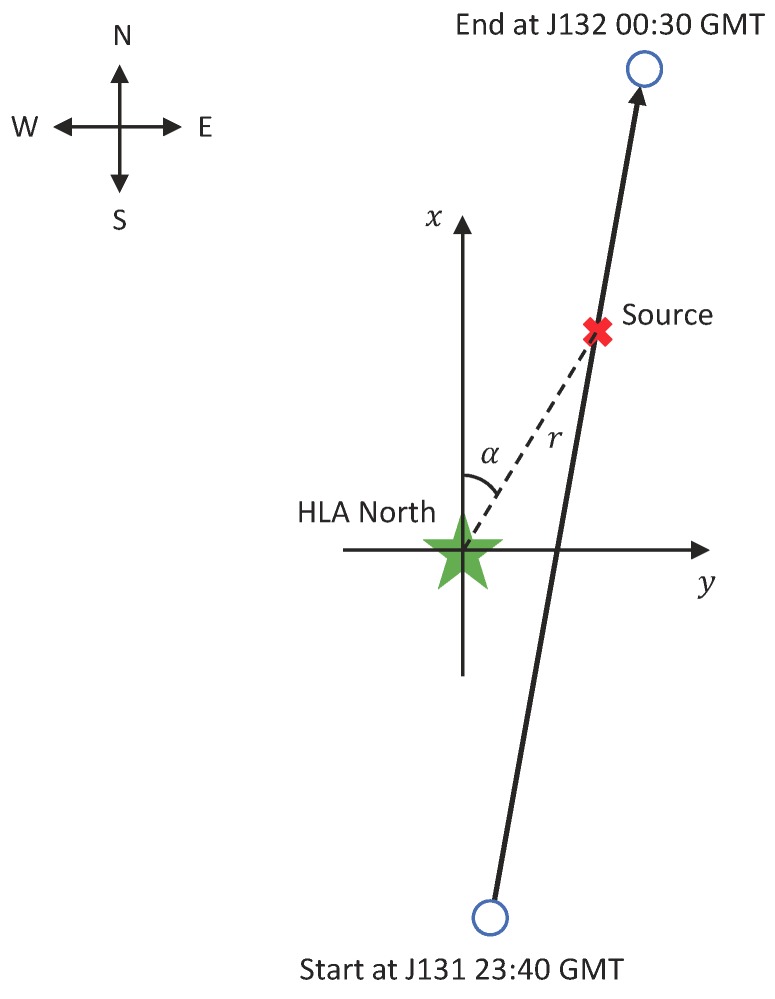
Map of the source movement and the location of the hydrophone array.

**Figure 14:**
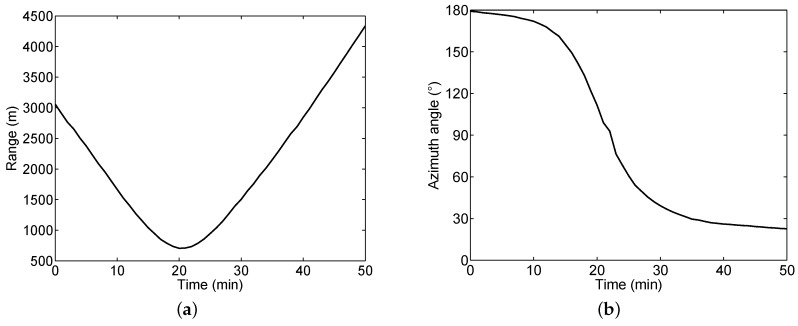
The ranges (**a**) and azimuth angles (**b**) between source and array from J131 23:40 GMT to J132 00:30 GMT.

The proposed method was further evaluated by real experimental data that were recorded by HLA North of SWellEx-96 Event S5. The water depth was 213 m and the HLA North array is a 240 m aperture horizontal array deployed on the seafloor. The source ship (R/V Sproul) started its track south of the array and proceeded northward at a speed of 5 knots. The signals of the deep source were used for processing. The map of the source movement and the location of the hydrophone array were shown in Figure 13. There were fifty minute signals from J131 23:40 GMT to J132 00:30 GMT that were recorded by HLA North (Day J131 corresponds to 5/10/96). The range and azimuth angle motions between source and array were plotted in Figure 14. To imitate the multi-source signals (i.e., a snapshot generated by several sources), we combined snapshots from the same source recorded at different positions. As a result, the NL of the resultant multi-source signal was higher than that in the original recordings, that is, the SNR was reduced when increasing the source number.

The experimental data with sample rate 3276.8 Hz were transformed to frequency by 4096-point FFT (Hanning windowed). The frame length was 1.25 s and the SCMs were averaged over 20 snapshots with 50% overlap. Considering the Doppler effect, processing frequencies were selected from three frequency bins centered on each of the nominal source frequencies. Accordingly, there were 3×F processing frequency bins if we took *F* source frequencies into account. Referring to Doppler Shift theory, the maximum Doppler shift is $\Delta f=\pm \frac{2.5}{1500}f_{i}=\pm 1.7\times 10^{-3}f_{i}$ ($f_{i}$ is the source frequency), which corresponds to ±0.083 to ±0.66 Hz for the pilot tones. Similar to Section 4.2, data augmentation is used to generate the training set (refer to Algorithm 1, $\vartheta =7^{\circ }$ and $\vartheta_{o}=0.5^{\circ }$).

### 5.2. Experimental Results

Firstly, we investigated the performance of our proposed method using different frequency bins in the two-source scenarios. The two-source signals were the combination of snapshots from J131 23:47 GMT to J131 23:53 GMT and snapshots from J132 00:19 GMT to J132 00:25 GMT, which were six minutes in total. Three frequency bin sets were investigated, which were $\left\{49\;64\;79\;94\;112\;130\;148\;166\;201\;235\;283\;338\;388\right\}$ Hz, $\left\{94\;112\;130\;148\;166\;201\;235\;283\;338\;388\right\}$ Hz, and $\left\{49\;94\;148\;235\;283\;338\right\}$ Hz (i.e., 3×13, 3×10, and 3×6 frequency bins used for processing because of Doppler shift). The parameters of DNNs in direction finding and source ranging were set the same as those in the simulations, while the input dimensions were slightly different from those in the simulation because of the difference in the number of frequency bins.

**Figure 15:**
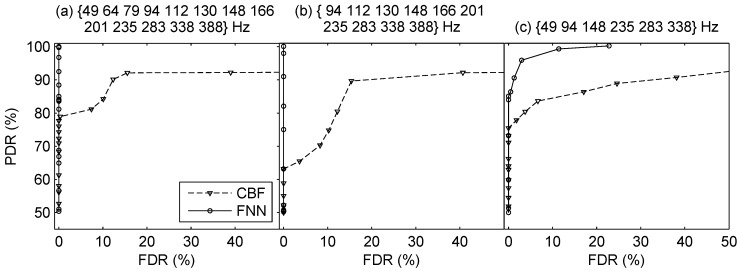
ROC curves of direction finding using different frequency bins in the two-source scenario using the real experimental data.

**Figure 16:**
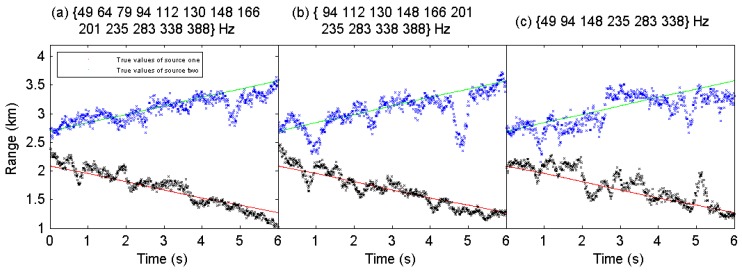
The source rang estimates across time using different frequency bins in the two-source scenario using the real experimental data.

In direction finding, the ROC curves are plotted in Figure 15. The results show that the proposed direction finding method outperforms CBF significantly. The FNN can detect more sources effectively while having lower false detection relative to CBF. Also, the lower threshold $\eta_{o}$ means the strong ability to suppress interferences. As there are more phantom peaks of CBF, its FDRs are much higher than FNN. The ${\mathrm{MAE}}_{\alpha }$, ${\mathrm{MAE}}_{r}$, ${\mathrm{MRE}}_{r}$, the corresponding $\eta_{o}$, PDR and FDR are summarized in Table 6. The proposed method achieves the best performance in all conditions. Besides, the source range estimates across time are plotted in Figure 16, where the results using the three sets of frequency bins are respectively shown in Figure 16a–c. We can see that the proposed method can give reliable estimates of the range of two sources successively, although the performance degrades with reduction of the frequency bins.

To demonstrate that LSTM-RNN can make full advantage of the long-term temporal contextual information, we compared the FNN with LSTM-RNN for source ranging. The results are also shown in Table 6. It can be seen that the LSTM-RNN outperforms FNN, especially when the number of frequency bins decreases. The results reveal the superiority of LSTM-RNN on modeling the long-term information.

Next, we investigated the influence of the parameters of LSTM-RNN on the performance of source ranging. Thirteen source frequencies were used (39 bins). The hidden layers were changed from 2 to 4, the hidden nodes were set to 512, 896, and 1024, and the learning rates were chosen from 0.0005, 0.001, and 0.002. The testing results are summarized in Table 7. The best results were achieved by the network with 3 hidden layer, 896 hidden nodes, and learning rate 0.001. From the results, generally, the change in parameters has little influence on the performance of source ranging.

**Figure 17:**
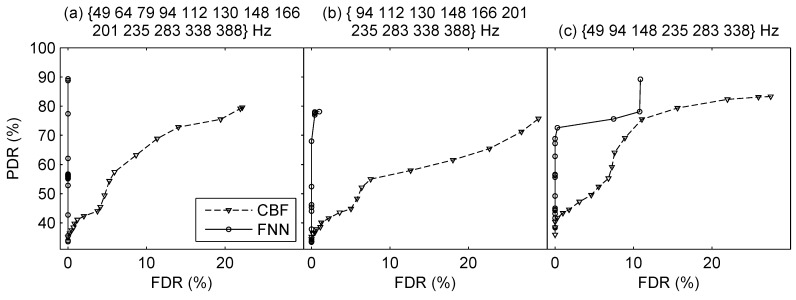
ROC curves of direction finding using different frequency bins in the three-source scenario using the real experimental data.

Finally, we evaluated the proposed method on the three-source scenario. The three-source signals contained six minutes that were combined by snapshots from J131 23:47 GMT to J131 23:53 GMT, snapshots from J132 00:07 GMT to J132 00:13 GMT and snapshots from J132 00:23 GMT to J132 00:29 GMT. The ROC curves are plotted in Figure 17 and the ${\mathrm{MAE}}_{\alpha }$, ${\mathrm{MAE}}_{r}$, ${\mathrm{MRE}}_{r}$, and the corresponding PDR and FDR are summarized in Table 6. The threshold $\eta_{o}$ is the same as the two-source scenario. From the results, we can find the proposed method generally outperforms the competing methods. Also, the LSTM-RNN exhibits a more robust performance than FNN.

## 6. Conclusions

This paper presents a two-stage DNN based method for multiple source localization in a shallow water environment using UHA. We attempt to train a general and flexible model using single-source signals that is suitable for source ranging in various scenarios with different source numbers. The subarray beamforming technique is taken as the feature extractor that separate sources at the level of feature and LSTM-RNN is leveraged for source ranging. Since the subarray beamforming requires the direction information to be known beforehand, a FNN model is trained for direction finding, meanwhile determine the source number. Both the simulation and experimental results demonstrate the effectiveness and superiority of the proposed framework. As LSTM-RNN can make full use of long-term temporal contextual information for the current estimation, it is an ideal model for source ranging. Our method can localize arbitrary numbers of sources that overlap in the TF domain. In our future work, we will make further efforts to improve the robustness of the proposed method in the more complex environments with lower SNRs and more sources.

## Figures and Tables

**Table 1:** Algorithm 1: data augmentation process.

**Input:** original data Φ;
**Output:** augmented training set Ψ;
Set Ψ=⌀;
**For** each sample $Y^{\kappa }\left(f_{i}\right)$ in Φ **do**
**For** offset $\alpha_{\zeta }=-\vartheta :\vartheta_{o}:\vartheta$ **do**
Add $\alpha_{\zeta }$ to the true azimuth $\hat{\alpha }$, $\alpha^{\prime }=\hat{\alpha }+\alpha_{\zeta }$;
Generate the beamformed signals using Equation (17);
Generate feature $u_{\zeta }^{\kappa }$ using Equation (9);
$\Psi =\Psi ⋃u_{\zeta }^{\kappa }$;
**End**
**End**

**Table 2:** The performance comparison under different SNRs in the two-source and three-source scenarios using the simulated data on HCA.

	SNR (dB)	Method	$\eta_{o}$	MAE${}_{\alpha }$ (degree)	PDR (%)	FDR (%)	MAE${}_{r}$ (km)	MRE${}_{r}\;\left(\%\right)$
Two sources	15	FNN+LSTM-RNN	**0.1**	**0.24**	**100.0**	**0.0**	**0.08**	**3.2**
		FNN+FNN					0.43	16.0
		CBF	0.25	0.26	**100.0**	**0.0**	—	—
	5	FNN+LSTM-RNN	**0.1**	**0.24**	**100.0**	**0.0**	**0.09**	**3.4**
		FNN+FNN					0.57	22.2
		CBF	0.45	0.28	100.0	0.05	—	—
	−5	FNN+LSTM-RNN	**0.2**	**0.25**	**100.0**	**0.0**	**0.59**	**21.3**
		FNN+FNN					0.76	28.7
		CBF	0.55	0.53	82.4	20.8	—	—
Three sources	15	FNN+LSTM-RNN	**0.1**	**0.25**	**100.0**	**0.0**	**0.18**	**7.0**
		FNN+FNN					0.66	25.6
		CBF	0.3	0.29	**100.0**	**0.0**	—	—
	5	FNN+LSTM-RNN	**0.1**	**0.25**	**100.0**	**0.0**	**0.32**	**12.0**
		FNN+FNN					0.71	27.7
		CBF	0.35	0.31	99.9	0.7	—	—
	−5	FNN+LSTM-RNN	**0.1**	**0.27**	**100.0**	**0.0**	**0.74**	**28.8**
		FNN+FNN					0.81	31.9
		CBF	0.5	0.66	83.6	13.6	—	—

**Table 3:** The performance comparison under different SNRs in the two-source and three-source scenarios using the simulated data on HLA.

	SNR (dB)	Method	$\eta_{o}$	MAE${}_{\alpha }$ (degree)	PDR (%)	FDR (%)	MAE${}_{r}$ (km)	MRE${}_{r}\;\left(\%\right)$
Two sources	15	FNN+LSTM-RNN	**0.15**	**1.37**	100.0	1.5	**0.04**	**1.6**
		FNN+FNN					0.51	19.9
		CBF	0.6	1.79	**100.0**	**0.0**	—	—
	5	FNN+LSTM-RNN	**0.1**	**1.39**	**100.0**	**0.0**	**0.06**	**2.1**
		FNN+FNN					0.53	20.5
		CBF	0.7	1.79	100.0	2.3	—	—
	−5	FNN+LSTM-RNN	**0.1**	**1.49**	**100.0**	**0.0**	**0.67**	**25.8**
		FNN+FNN					0.71	27.7
		CBF	0.9	1.69	99.4	2.6	—	—
Three sources	15	FNN+LSTM-RNN	**0.1**	**1.52**	100.0	0.2	**0.15**	**5.8**
		FNN+FNN					1.00	38.5
		CBF	0.65	2.06	**100.0**	**0.0**	—	—
	5	FNN+LSTM-RNN	**0.1**	**1.55**	**100.0**	**0.0**	**0.22**	**8.1**
		FNN+FNN					0.98	38.8
		CBF	0.7	2.06	100.0	0.0	—	—
	−5	FNN+LSTM-RNN	**0.1**	**1.61**	**100.0**	**0.0**	**0.65**	**24.0**
		FNN+FNN					1.04	38.9
		CBF	0.9	2.00	99.6	4.7	—	—

**Table 4:** ${\mathrm{MAE}}_{r}$ and ${\mathrm{MRE}}_{r}$ comparison when two SLs are different on HCA.

ΔSL (dB)	${\mathrm{MAE}}_{r}$ (km)	${\mathrm{MRE}}_{r}$ (%)
2	0.19	6.7
4	0.27	9.8
6	0.32	12.4

**Table 5:** ${\mathrm{MAE}}_{r}$ and ${\mathrm{MRE}}_{r}$ comparison under different source separations on HCA.

Separation (Degree)	${\mathrm{MAE}}_{r}$ (km)	${\mathrm{MRE}}_{r}$ (%)
2	0.03	1.4
3	0.04	1.9
5	0.03	2.1
7	0.03	2.1
10	0.03	2.2

**Table 6:** The performance comparison with different frequency bins in the two-source and three-source scenarios using the real experimental data.

	Frequency (Hz)	Method	$\eta_{o}$	MAE${}_{\alpha }$ (degree)	PDR (%)	FDR (%)	MAE${}_{r}$ (km)	MRE${}_{r}\left(\%\right)$
Two sources	{49647994112130148 166201235283338388}	FNN+LSTM-RNN	0.1	**2.74**	**100.0**	**0.0**	**0.11**	**5.0**
		FNN+FNN					0.14	5.6
		CBF	0.3	3.49	90.1	12.3	—	—
	{94112130148166201 235283338388}	FNN+LSTM-RNN	0.1	**3.32**	**100.0**	**0.0**	**0.13**	**5.4**
		FNN+FNN					0.18	7.9
		CBF	0.25	3.34	89.6	15.4	—	—
	$\left\{49\;94\;148\;235\;283\;338\right\}$	FNN+LSTM-RNN	0.2	**3.35**	**95.9**	**3.0**	**0.15**	**6.7**
		FNN+FNN					0.24	10.2
		CBF	0.3	3.44	86.4	17.1	—	—
Three sources	{49647994112130148 166201235283338388}	FNN+LSTM-RNN	0.1	**3.34**	**89.4**	**0.0**	**0.36**	**15.6**
		FNN+FNN					0.47	23.7
		CBF	0.15	3.42	79.2	22.0	—	—
	{94112130148166201 235283338388}	FNN+LSTM-RNN	0.1	3.84	**78.1**	**1.0**	**0.34**	**14.0**
		FNN+FNN					0.55	25.2
		CBF	0.15	**3.24**	71.2	26.8	—	—
	$\left\{49\;94\;148\;235\;283\;338\right\}$	FNN+LSTM-RNN	0.1	3.57	**89.2**	**10.9**	**0.41**	**19.3**
		FNN+FNN					0.51	24.8
		CBF	0.15	**3.55**	82.3	22.0	—	—

**Table 7:** ${\mathrm{MAE}}_{r}$ and ${\mathrm{MRE}}_{r}$ comparison with different parameters of LSTM-RNN using the experimental data.

Parameter			${\mathrm{MAE}}_{r}$	${\mathrm{MRE}}_{r}$
Hidden Layer	Hidden Node	Learning Rate		
3	512	0.001	0.16	6.4%
3	896	0.001	**0.11**	**5.0**%
3	1024	0.001	0.14	5.9%
3	896	0.0005	0.13	5.2%
3	896	0.002	0.13	5.2%
2	896	0.001	0.12	5.0%
4	896	0.001	0.13	5.4%

## Abbreviations

The following abbreviations are used in this manuscript:

DNN	Deep neural network
MFP	Matched-field processing
ML	Maximum likelihood
CS	Compressive sensing
MAP	maximum a posteriori
SBL	Sparse Bayesian learning
UHA	Underwater horizontal arrays
TF	Time-frequency
DOA	Direction of arrival
FNN	Feed-forward neural network
LSTM-RNN	Long short term memory - recurrent neural network
CBF	Conventional beamforming
SCM	Spatial correlation matrix
BP	Back propagation
TDNN	Time delay neural network
MSE	Mean square error
SSP	Sound speed profile
HCA	Horizontal circular array
HLA	Horizontal line array
FFT	Fast Fourier transformation
ReLU	Rectified linear units
PDR	Positive detection rate
FDR	False detection rate
ROC	Receiver operating performance characteristics
MAE	Mean absolute error
MRE	Mean relative error
SNR	Signal-to-noise ratio
SL	Source level
NL	Noise level
